# Skeletal muscle and visceral adipose radiodensities are pre‐surgical, non‐invasive markers of aggressive kidney cancer

**DOI:** 10.1002/jcsm.13429

**Published:** 2024-01-24

**Authors:** Helena Furberg, Patrick T. Bradshaw, Andrea Knezevic, Linnea Olsson, Stacey Petruzella, Emily Stein, Mike Paris, Jessica Scott, Oguz Akin, A. Ari Hakimi, Paul Russo, Alejandro Sanchez, Bette Caan, Marina Mourtzakis

**Affiliations:** ^1^ Department of Epidemiology and Biostatistics Memorial Sloan Kettering Cancer Center New York NY USA; ^2^ Division of Epidemiology, School of Public Health University of California Berkeley Berkeley CA USA; ^3^ Department of Epidemiology University of North Carolina Chapel Hill NC USA; ^4^ Department of Kinesiology University of Waterloo Waterloo Canada; ^5^ Department of Medicine Memorial Sloan Kettering Cancer Center New York NY USA; ^6^ Department of Radiology Memorial Sloan Kettering Cancer Center New York NY USA; ^7^ Huntsman Cancer Institute Salt Lake City UT USA; ^8^ Department of Epidemiology Kaiser Permanente Oakland CA USA

**Keywords:** Body composition, Grade, Kidney cancer, Radiodensity, Skeletal muscle, Stage, Visceral adiposity

## Abstract

**Introduction:**

Most studies on body composition in kidney cancer have been conducted among patients with metastatic disease. Given that aggressive tumours can adversely impact body composition and even non‐metastatic tumours can be aggressive, we evaluated associations between pre‐surgical body composition features and tumour pathological features in patients with non‐metastatic clear cell renal cell cancer (ccRCC).

**Methods:**

The Resolve Cohort consists of 1239 patients with non‐metastatic ccRCC who underwent nephrectomy at Memorial Sloan Kettering Cancer Center between 2000 and 2020. The cross‐sectional areas and radiodensities of skeletal muscle, visceral adipose, and subcutaneous adipose tissues were determined from pre‐surgical computed tomography (CT) scans at the third lumbar vertebrae using Automatica software. Pearson's correlation coefficients describe inter‐relationships among BMI and body composition variables, while odds ratios (OR) and 95% confidence intervals (CI) estimate associations between continuous body composition features (per 1‐standard deviation) and advanced stage (Stage III vs. Stages I–II) and high Fuhrman grade (Grades 3–4 vs. 1–2) from multivariable logistic regression models that considered the potential impact of biological sex, contrast enhanced CTs, and early age at onset of ccRCC.

**Results:**

The cohort was predominantly male (69%), white (89%), and had a median age of 58. The proportion of patients presenting with advanced stage and high‐grade disease were 31% and 51%, respectively. In models that adjusted for demographics and all body composition variables simultaneously, decreasing skeletal muscle radiodensity (i.e., more fat infiltration) but increasing visceral adipose tissue radiodensity (i.e., more lipid depletion) were associated with advanced tumour features. Per 8.4 HU decrease in skeletal muscle radiodensity, the odds of presenting with advanced stage was 1.61 (95% CI: 1.34–1.93). Per 7.22 HU increase in visceral adipose tissue radiodensity, the odds of presenting with advanced stage was 1.45 (95% CI: 1.22–1.74). Skeletal muscle index (i.e., sarcopenia) was not associated with either tumour feature. Similar associations were observed for Fuhrman grade, a more direct marker of tumour aggressiveness. Associations did not differ by sex, contrast use, or age at onset of ccRCC.

**Conclusions:**

Lipid infiltrated skeletal muscle, but lipid depleted visceral adipose tissue were independently associated with advanced tumour features in non‐metastatic ccRCC. Findings highlight the importance of evaluating the full range of body composition features simultaneously in multivariable models. Interpreting pre‐surgical CTs for body composition for patients may be a novel and non‐invasive way to identify patients with aggressive renal tumours, which is clinically relevant as renal biopsies are not routinely performed.

## Introduction

Clear cell renal cell carcinoma (ccRCC) is the most common and lethal type of kidney cancer. Although high body mass index (BMI) is an established risk factor for developing the disease, it exhibits a counterintuitive association with clinical outcomes where patients with ccRCC who are overweight or obese at the time of surgery survive longer than patients who are classified as normal weight.^1^ Molecular studies by our group and others have shown that the survival advantage of patients with overweight or obesity may be related to their tumours being less aggressive.^2^, ^3^, ^4^, ^5^ Additional insights are being gained by interpreting body composition variables from computed tomography (CT) scans and examining how they are related to clinical outcomes. CTs allow for estimation for both the quantity (i.e., cross‐sectional area) and quality (i.e., radiodensity) of skeletal muscle, visceral adipose, and subcutaneous adipose tissues. However, most research to date has been conducted among patients with metastatic RCC where low skeletal muscle mass and radiodensity have emerged as adverse prognostic factors.^6^, ^7^ In the metastatic setting, these markers of poor muscle health likely reflect cancer cachexia, defined as an involuntary loss of muscle mass with or without accompanying adipose tissue depletion, which is driven by a constellation of factors including aging, systemic treatment, anorexia, physical inactivity, and aggressive tumour biology.^8^

Determining the clinical relevance of body composition in the non‐metastatic setting is important because cachexia can develop progressively from pre‐cachexia to refractory cachexia at any time during the disease course, and even early stage ccRCC can be aggressive. Non‐invasive markers of aggressive tumour biology are needed for to help risk stratify the increasing number of patients presenting with renal masses as renal biopsies are not routinely performed and active surveillance may be appropriate for patients with indolent tumours. To date, prior studies conducted among patients with non‐metastatic disease have focused primarily on candidate body composition variables, such as skeletal muscle or visceral adipose tissue, with inconsistent findings.^9^, ^10^, ^11^, ^12^, ^13^, ^14^, ^15^, ^16^, ^17^ Notably, no prior study has simultaneously evaluated the area and the radiodensity of skeletal muscle, visceral adiposity, and subcutaneous adiposity, which is needed to identify which aspect(s) of body composition are most clinically relevant. We established the Resolve Study at Memorial Sloan Kettering Cancer Center (MSK) in 2019 to conduct the first large scale molecular epidemiology study to determine the clinical relevance and molecular underpinnings of body composition features in patients with non‐metastatic ccRCC treated by nephrectomy. In the current report, we examined how the mass and radiodensity of skeletal muscle, visceral adipose tissue, and subcutaneous adipose tissues from pre‐surgical CT scans are associated with BMI, with each other, and with pathological tumour features. We hypothesized that poor muscle health (i.e., low skeletal muscle mass and low skeletal muscle radiodensity) may be markers of subclinical wasting driven by tumour aggressiveness and therefore be associated with advanced stage and high grade, independent of visceral and subcutaneous adiposity variables.

## Methods

### Study cohort

The Resolve Study is a retrospective cohort study funded by the National Cancer Institute (PI: Furberg CA233885) and approved by the MSK Institutional Review Board. This study has therefore been performed in accordance with the ethical standards laid down in the 1964 Declaration of Helsinki and its later amendments and all relevant state laws. Patients were eligible for the Resolve Study if they were between ages of 18 and 85 when they underwent nephrectomy at MSK for histologically confirmed non‐metastatic (Stages I–III) clear cell renal cell carcinoma (ccRCC) between January 2000 and March 2020. Eligible patients had no prior personal history of cancer and received no neoadjuvant systemic treatment. Of these 1919 patients with ccRCC, we excluded 680 (35%) patients who did not have a CT scan taken <3 months prior to surgery. The median time from CT scan to surgical date was 28 days (interquartile range 20–48 days). The final analytic cohort was comprised of *n* = 1239 patients with non‐metastatic ccRCC. To assess selection bias, we compared demographic, BMI, and clinical variable distributions between patients who were included and excluded based on CT eligibility. Patients included in our Resolve Study were slightly younger (median age 58 vs. 60, *P* < 0.0001) and had a higher proportion of high‐grade disease (61% vs. 49%, *P* < 0.001).

### Body composition assessment

A single slice CT image at the third lumbar vertebrate was identified for each patient and segmented by a trained analyst blinded to clinical parameters and patient outcomes (E.S.). The open‐source program AUTOMATICA^18^ was used to characterize skeletal muscle (psoas, erector spinae, quadratus lumborum, transverse abdominus, rectus abdominus, as well as internal and external obliques) and adipose tissue (visceral and subcutaneous) depots using Hounsfield unit (HU) threshold values: −29 to +150 for skeletal muscle, −150 to −50 for visceral adipose tissue, and −190 to −30 for subcutaneous adipose tissue.^19^ Segmentation resulted in continuous values of six body composition variables: cross‐sectional area (CSA; cm^2^) and radiodensity (HU) of skeletal muscle, visceral adipose, and subcutaneous adipose tissues. Each CSA was divided by height in meters squared and resulted in an index for each tissue type (i.e., tissue quantity standardized for height). Radiodensity of muscle tissue (i.e., tissue quality) is regarded as a radiologic marker of lipid infiltration with lower radiodensities reflecting higher lipid content.^20^ Low skeletal muscle radiodensity, known as myosteatosis, compromises muscle function and disrupts metabolism.^21^ Conversely, higher adipose tissue radiodensity is correlated with smaller adipocytes with lower lipid content, and can reflect inflammation, fibrosis, vascularity, or metabolic changes.^22^ It is important to note that lower radiodensity values of skeletal muscle, but higher adipose tissue radiodensities are considered pathological. We noted whether intravenous contrast was present on the segmented CT because our group previously showed the use of contrast can influence radiodensity values and result in misclassification.^23^

### Clinical data

The following information was available for each patient from a prospectively maintained clinical database at MSK: demographics (age, biological sex, and race), smoking history (ever vs. never), co‐morbidities (i.e., lifetime existence of and/or treatment for diabetes and/or hypertension). Also available was a variable that captured whether the tumour was detected incidentally or through symptoms, the latter of which included unanticipated weight loss prior to surgery. Height and weight were measured during the pre‐surgical visit and categorized into BMI using the formula weight divided by height squared. BMI was evaluated as a continuous variable and in the WHO categories of normal weight (BMI < 25 kg/m^2^), overweight (25 ≤ BMI < 30 kg/m^2^), and obese (BMI ≥ 30 kg/m^2^). Tumour characteristics were abstracted from pathology reports and included tumour size, lymph node involvement, metastatic status, Fuhrman grade for patients diagnosed before January 1, 2018 (*n* = 1125), and the International Society of Urological Pathology (ISUP) grade for patients diagnosed after this date (*n* = 114).

### Statistical analyses

All statistical analyses were performed with the R statistical software, version 4.2.2 (https://www.R‐project.org/). We generated descriptive statistics for all variables with medians and inter‐quartile ranges for continuous variables and counts and percentages for categorical variables. Sex‐stratified analyses are presented given the known differences in body composition variables between males and females. We calculated pairwise Pearson correlation coefficients, bivariate scatterplots, and univariate kernel density plots for the six body composition variables under consideration. We estimated odds ratios (OR) and 95% confidence intervals (CI) with logistic regression for the association between the body composition variables and the primary outcomes of advanced stage (Stage III vs. I or II) and advanced Fuhrman grade (Grade 3 or 4 vs. 1 or 2). We first assessed nonlinear trends in body composition, by comparing models with variables coded linearly to restricted quadratic spline coding using the Akaike Information Criterion (AIC)^24^, ^25^ with knots at the 10th, 25th, 75th, and 90th percentiles and adjusted for age. Given no evidence for nonlinearity by AIC model selection, we present estimates with body composition expressed linearly in the log‐odds, scaled to 1‐standard deviation (SD) changes based on the distribution of each body composition measure in the overall population. This scaling was chosen to yield a consistent and meaningful magnitude of change in each exposure across the models considered. We present unadjusted estimates for individual models of body composition variables as well as age‐ and sex‐adjusted estimates for models with individual and simultaneous inclusion of the body composition variables. We present results stratified by biological sex (male/female) and early age at onset of ccRCC based on age 46^26^ with corresponding 1‐degree of freedom test for effect measure modification. To explore the impact of contrast enhanced CTs on our results, we first performed a sensitivity analysis where we excluded patients whose CTs contained contrast (*n* = 217, 17%); next we tested whether associations between body composition and pathological tumour features differed by contrast using effect measure modification, and finally, present multivariable models adjusted for contrast. We conducted an exploratory analysis of the potential impact of the tumour grade classification change by performing a probabilistic sensitivity analysis following methods for outcome misclassification^27^ based on the concordance between Fuhrman and ISUP grade reported by Odeh et al.^28^ For methodological details, see supporting information. All statistical tests used a significance level of 0.05.

## Results

The overall Resolve Cohort was predominantly male (69%), white (89%), and had a median age of 58 [interquartile range (IQR) 49–66 years] (Table 1). Sixty four per cent had Stage I, 5% had Stage II, and 31% had advanced stage (Stage III) disease. The distribution of Fuhrman grades 1 through 4 were 2%, 47%, 43%, and 8%, respectively. Most patients underwent partial nephrectomy (63%). While demographic factors were similarly distributed by sex, higher proportions of males than females were diagnosed with advanced stage (33% vs. 27%) and high grade (58% vs. 35%) tumours. Males were more likely than females to be detected through symptoms (29% vs. 20%) and have slightly larger tumours (4.0 cm vs. 3.6 cm). Table 2 demonstrates considerable differences in body composition by sex, despite males and females having similar median BMIs (~29 kg/m^2^). Males had higher median values of skeletal muscle index and density as well as visceral adipose tissue index, but lower median values of subcutaneous adipose tissue index than females. Median values of adiposity‐related radiodensities were similar by sex.

**Table 1:** Demographic and clinical characteristics of ccRCC patients in the Resolve Cohort overall (*n* = 1239) and stratified by sex

Variable	Overall (*n* = 1239)	Males (*n* = 851)	Females (*n* = 388)
Age in years^a^	58 (49, 66)	57 (49, 65)	60 (50, 68)
Race			
White	1103 (89%)	758 (89%)	345 (89%)
Asian	55 (4.4%)	38 (4.5%)	17 (4.4%)
Black	36 (2.9%)	22 (2.6%)	14 (3.6%)
Native American/Other	17 (1.4%)	13 (1.5%)	4 (1.0%)
Refused/Unknown	28 (2.3%)	20 (2.4%)	8 (2.1%)
Stage			
Stage I	795 (64%)	524 (61.6%)	271 (69.8%)
Stage II	63 (5%)	49 (5.8%)	14 (3.6%)
Stage III	381 (31%)	278 (32.6%)	103 (26.5%)
Fuhrman grade			
Grade 1	27 (2.4%)	15 (2.0%)	12 (3.3%)
Grade 2	534 (47%)	310 (41%)	224 (62%)
Grade 3	479 (43%)	372 (49%)	107 (30%)
Grade 4	85 (7.6%)	66 (8.7%)	19 (5.2%)
Missing	114	88	26
Tumour size, cm^a^	4.00 (2.50, 6.00)	4.00 (2.70, 6.40)	3.60 (2.50, 5.43)
Detection method			
Incidental	914 (74%)	604 (71%)	310 (80%)
Symptomatic	325 (26%)	247 (29%)	78 (20%)
Surgery type			
Partial nephrectomy	775 (63%)	525 (62%)	250 (64%)
Radical nephrectomy	464 (37%)	326 (38%)	138 (36%)
Smoking status			
Current smoker	168 (14%)	33 (8.5%)	135 (16%)
Former smoker	436 (35%)	120 (31%)	316 (37%)
Never smoker	635 (51%)	235 (61%)	400 (47%)
Lifetime history of diabetes	193 (16%)	131 (15%)	62 (16%)
Lifetime history of hypertension	674 (54%)	467 (55%)	207 (53%)
Contrast enhanced CT	217 (18%)	149 (18%)	68 (18%)

^a^ Median and interquartile range (IQR).

**Table 2:** Body size characteristics of ccRCC patients overall and stratified by sex

Variable^a^	Overall (*n* = 1239)	Males (*n* = 851)	Females (*n* = 388)
Body mass index^b^	29.6 (26.4, 33.4)	29.6 (26.7, 33.1)	29.4 (25.8, 34.9)
Skeletal muscle index^c^	54 (46, 61)	57 (52, 64)	45 (40, 50)
Skeletal muscle density^d^	38 (32, 43)	40 (34, 45)	33 (28, 40)
Visceral adipose tissue index^c^	64 (41, 90)	71 (49, 95)	48 (27, 69)
Visceral adipose tissue density^d^	−96 (−100, −91)	−97 (−101, −92)	−95 (−99, −89)
Subcutaneous adipose tissue index^c^	73 (52, 105)	65 (46, 89)	101 (71, 143)
Subcutaneous adipose tissue density^d^	−101 (−105, −97)	−101 (−105, −96)	−103 (−106, −99)

^a^ Median and interquartile range.

^b^ kg/m^2^.

^c^ cm^2^/m^2^.

^d^ Hounsfield units.

Table 3 presents the pairwise Pearson Correlation coefficients among body size variables separately for males (3a) and females (3b). The strongest correlations observed in both sexes were between BMI and subcutaneous adipose tissue index (*r* = 0.8), followed by BMI and visceral adipose tissue index (*r* = 0.65). BMI was moderately correlated with skeletal muscle index in females (*r* = 0.65) but less so in males (*r* = 0.52). We detected a moderate positive correlation between visceral adipose tissue density and subcutaneous adipose tissue density, (*r* = 0.66), and a moderate negative correlation between visceral adipose tissue density and visceral adipose tissue index (*r* = −0.68). The remaining body composition variables showed weak correlations in both males and females. Figures S1 and S2 show more detailed correlation matrices for males and females, respectively.

**Table 3:** Pearson correlation coefficients among BMI and body composition variables in males (a) and females (b)

	BMI	SMI	SMD	VATI	VATD	SATI	SATD
**a. Males (*n* = 851)**								
BMI	1						
SMI	0.52	1					
SMD	−0.38	0.26	1				
VATI	0.66	0.26	−0.43	1			
VATD	−0.35	−0.12	0.29	−0.66	1		
SATI	0.82	0.23	−0.43	0.45	−0.29	1	
SATD	−0.24	0.04	0.28	−0.31	0.70	−0.38	1
**b. Females (*n* = 388)**							
BMI	1						
SMI	0.65	1					
SMD	−0.34	0.12	1				
VATI	0.66	0.39	−0.51	1			
VATD	−0.41	−0.15	0.41	−0.68	1		
SATI	0.89	0.50	−0.34	0.53	−0.41	1	
SATD	−0.15	0.06	0.25	−0.22	0.66	−0.29	1

BMI, body mass index; SATD, subcutaneous adipose tissue density; SATI, subcutaneous adipose tissue index; SMD, skeletal muscle density; SMI, skeletal muscle index; VATD, visceral adipose tissue density; VATI, visceral adipose tissue index.

Table 4 presents unadjusted and adjusted ORs for each body composition variable in relation to advanced stage and high grade for males and females combined. Although our formal test for effect measure modification by sex was not significant for any comparison (all *P*‐values ≥0.22), we present results separately for males (*n* = 698) and females (*n* = 334) to facilitate comparison to prior studies (Table 5). Decreasing skeletal muscle density was associated with advanced stage in fully adjusted models that accounted for age, and all other body composition variables. Per 8.4 HU decrease in skeletal muscle density, the odds of presenting with advanced stage disease increased by 61% [OR 1.61 (95% CI: 1.34–1.93)]. Although decreasing skeletal muscle index (i.e., muscle quantity) was initially associated with advanced stage [OR 1.13 (95% CI: 1.00–1.28)], it was attenuated and not significant after multivariable adjustment [OR 1.09 (95% CI: 0.90–1.32)]. Visceral adipose tissue density was positively associated with advanced stage in adjusted models that accounted for age, and all other body composition variables. Per 7.2 HU increase in visceral adipose tissue density, the odds of presenting with advanced stage disease increased by 41% (OR 1.41 (95% CI: 1.10–1.81). Similar patterns of association were observed between body composition variables and high Fuhrman grade. Per 8.4 HU decrease in skeletal muscle density, the odds of presenting with high grade increased by 54% [OR 1.54 (95% CI: 1.28–1.86)]. Per 7.2 HU increase in visceral adipose tissue density, the odds of presenting with high grade increased by 36% (OR 1.36 (95% CI: 1.07–1.74)). The impact of contrast enhanced CTs and age at onset are shown in Tables S1 and S2, respectively; neither resulted in any material changes to any of the ORs. Results from the exploratory probabilistic sensitivity analysis that reclassified Fuhrman grade into ISUP grade also resulted in qualitatively similar, but slightly attenuated associations as shown in Supplemental Table 3. After reclassification, the association with skeletal muscle radiodensity remained notable [corrected estimated OR 1.25 (95% CI: 1.25 (1.01–1.55)], as did the association with visceral adipose tissue radiodensity [corrected estimated OR 1.29 (95% CI: 0.97–1.75)]. As expected, the confidence intervals are slightly wider for the sensitivity analyses as the uncertainty in the re‐classification step is propagated through these estimates.

**Table 4:** Associations between each body composition variable and advanced pathological characteristics in the Resolve Cohort

	Unadjusted OR (95% CI)	Age‐ and sex‐adjusted OR (95% CI)	Fully adjusted^a^ OR (95% CI)
Stage (Stage 3 vs. Stages 1–2), *n* = 1239			
SMI per −10.99 cm^2^/m^2^	1.13 (1.00–1.28)	1.15 (0.98–1.36)	1.09 (0.90–1.32)
SMD per −8.40 HU	1.53 (1.35–1.73)	1.51 (1.31–1.75)	**1.61 (1.34–1.93)**
SATI per 46.57 cm^2^/m^2^	1.01 (0.90–1.14)	1.11 (0.97–1.26)	0.92 (0.76–1.10)
SATD per 7.22 HU	1.02 (0.90–1.15)	0.97 (0.86–1.10)	0.90 (0.74–1.10)
VATI per 35.26 cm^2^/m^2^	1.20 (1.06–1.35)	1.07 (0.94–1.22)	1.19 (0.95–1.50)
VATD per 7.61 HU	1.02 (0.90–1.15)	1.05 (0.93–1.19)	**1.41 (1.10–1.81)**
Fuhrman grade (Grades 3–4 vs. Grades 1–2), *n* = 1125			
SMI per −10.99 cm^2^/m^2^	0.92 (0.82–1.03)	1.13 (0.97–1.32)	1.04 (0.86–1.26)
SMD per −8.40 HU	1.24 (1.10–1.40)	1.40 (1.21–1.63)	**1.54 (1.28–1.86)**
SATI per 46.57 cm^2^/m^2^	0.88 (0.78–0.99)	1.06 (0.93–1.21)	0.93 (0.78–1.11)
SATD per 7.22 HU	1.08 (0.96–1.21)	0.99 (0.88–1.12)	0.91 (0.75–1.11)
VATI per 35.26 cm^2^/m^2^	1.20 (1.06–1.35)	1.00 (0.88–1.14)	1.10 (0.88–1.38)
VATD per 7.61 HU	1.00 (0.89–1.12)	1.08 (0.96–1.23)	**1.36 (1.07–1.74)**

OR (95% CI): Odds ratio and 95% confidence interval.

SATD, subcutaneous adipose tissue density; SATI, subcutaneous adipose tissue index; SMD, skeletal muscle density; SMI, skeletal muscle index; VATD, visceral adipose tissue density; VATI, visceral adipose tissue index.

* Adjusted for age, sex, and all body composition variables.

**Table 5:** Sex‐specific fully adjusted^a^ associations between body composition variables and advanced stage and high grade in the Resolve Cohort

	Fully adjusted OR (95% CI) among males (*n* = 851)	Fully adjusted OR (95% CI) among females (*n* = 388)	Interaction test *P*‐value
Stage (Stage 3 vs. Stages 1–2), *n* = 1239			
SMI per −10.99 cm^2^/m^2^	1.12 (0.91–1.39)	0.94 (0.61–1.47)	0.472
SMD per −8.40 HU	1.63 (1.31, 2.03)	1.68 (1.22, 2.35)	0.867
SATI per 46.57 cm^2^/m^2^	0.80 (0.62–1.04)	1.01 (0.77–1.32)	0.221
SATD per 7.22 HU	0.87 (0.69–1.11)	0.87 (0.60–1.25)	0.965
VATI per 35.26 cm^2^/m^2^	1.18 (0.91–1.54)	1.29 (0.81–2.05)	0.747
VATD per 7.61 HU	1.36 (1.00–1.86)	1.72 (1.09–2.74)	0.407
Fuhrman Grade (Grades 3–4 vs. Grades 1–2), *n* = 1125			
SMI per −10.99 cm^2^/m^2^	1.00 (0.81–1.23)	1.21 (0.78–1.88)	0.433
SMD per −8.40 HU	1.64 (1.31–2.07)	1.35 (0.78–1.32)	0.302
SATI per 46.57 cm^2^/m^2^	0.88 (0.68–1.14)	1.02 (0.70–1.40)	0.441
SATD per 7.22 HU	0.88 (0.69–1.13)	0.99 (0.68–1.34)	0.592
VATI per 35.26 cm^2^/m^2^	1.07 (0.82–1.42)	1.18 (0.78–1.85)	0.699
VATD per 7.61 HU	1.35 (0.99–1.84)	1.42 (0.93–2.17)	0.857

OR (95% CI): Odds ratio and 95% confidence interval.

Global test of all body composition‐sex interactions, stage analysis: Likelihood ratio test *P*‐value: 0.49.

Global test of all body composition‐sex interactions, grade analysis: Likelihood ratio test *P*‐value: 0.92.

SATD, subcutaneous adipose tissue density; SATI, subcutaneous adipose tissue index; SMD, skeletal muscle density; SMI, skeletal muscle index; VATD, visceral adipose tissue density; VATI, visceral adipose tissue index.

^a^ Adjusted for age, sex, and all body composition variables.

## Discussion

This is the first comprehensive investigation in patients with non‐metastatic ccRCC to examine how the index and radiodensity of skeletal muscle, visceral adipose tissue, and subcutaneous adipose tissues interpreted from pre‐surgical CT scans are related to BMI, each other, and pathological tumour features. Although males and females presented with similar median BMIs, they differed considerably in distributions of body composition. Despite these differences, correlations among body composition variables, and associations between each body composition variable and pathological tumour features were similar by sex. Lower skeletal muscle radiodensity (i.e., fat infiltration) and higher visceral adipose tissue radiodensity (i.e., lipid depletion) were independently associated with both advanced stage and higher grade ccRCC in multivariable models. Our findings demonstrate the importance of accounting for all body composition variables in one model and suggest that the quality as opposed to quantity of skeletal muscle and visceral adipose tissues may be novel and non‐invasive markers of tumour aggressiveness. This is clinically relevant as CTs could be interpreted prior to treatment to potentially identify higher risk patients who may need to be managed differently.

Radiodensities of skeletal muscle and visceral adipose tissue are regarded as radiologic markers of their lipid content with lower radiodensities reflecting higher lipid content. In our study, lower skeletal muscle density and higher visceral adipose tissue density were strongly and independently associated with advanced stage. Importantly, they were also associated with high grade, a more direct measure of tumour cell proliferation. Lower skeletal muscle density suggests fatty infiltration into the skeletal muscle, known as myosteatosis, which compromises muscle function and disrupts metabolism.^21^ Myosteatosis has been associated with increasing age, reduced muscle strength, higher rates of frailty, physical function impairments, and insulin resistance,^29^ all of which may help explain why recent systematic reviews and meta‐analyses found that lower skeletal muscle density is associated with shorter survival in patients with cancer^30^ and in the general population.^31^ Vrieling et al. reported that low skeletal muscle density is associated with poor overall survival among metastatic RCC patients treated with systemic therapy^6^; a suggestive association was observed in localized patients with RCC.^17^ Whether low skeletal muscle density is a cause, or a consequence of aggressive tumours is not known. It has been hypothesized that myosteatosis could stimulate tumour growth through an insulin resistance‐related pathway where inflammatory adipokines are secreted by adipocytes in the skeletal muscle tissue.^29^ Alternatively, there is evidence from pancreatic and non‐small cell lung cancer suggesting that tumours can secrete systemic factors that adversely impact muscle quantity and quality.^32^, ^33^

Higher visceral adipose tissue density, which reflects lipid depletion of adipocytes, is also considered a marker of metabolic dysfunction as it has been associated with adverse biomarkers and cardiometabolic risk, including higher blood pressure, insulin resistance, lower high‐density lipoprotein levels, and adipokines.^34^, ^35^ A systematic review and meta‐analysis in mixed cancer populations reported higher mortality for lower visceral adipose tissue density.^36^ It is notable that we only found a positive association between higher visceral as opposed to subcutaneous adipose tissue density and advanced pathological features, despite that the adiposity tissues showed a moderately strong correlation. In ccRCC, the tumour microenvironment is comprised of visceral adipose tissue that surrounds the kidney (i.e., perinephric fat), which is invaded in advanced stages of the disease. Tumours classified as stage III have grown into the perinephric or renal sinus fat and made direct contact with visceral adipocyte cells, which provides the opportunity for crosstalk and potentially lipid exchange. Indeed, several studies of ovarian, breast, and melanoma tumours demonstrate a sharing of nutrients between tumours and cells in the tumour microenvironment including the transfer of fatty acids from cancer‐associated adipocytes to tumour cells.^37^, ^38^, ^39^ Future studies are needed to determine the contribution of the local perinephric fat to cancer initiation and/or promotion.

Our study confirms that body composition variables provide more detailed information about body size than BMI alone.^40^ The correlation between BMI and quantities of visceral and subcutaneous adipose tissues were stronger than for BMI and quantity of skeletal muscle. Notably, BMI was not strongly correlated with radiodensity measures of these tissues, suggesting that BMI is not a reliable marker of tissue quality. Median BMIs were the same in men and women; however, we observed the expected sex differences in the distributions of body composition variables where males had higher amounts of skeletal muscle and visceral adipose tissue but lower amounts of subcutaneous adipose tissue than females. Despite these differences, the correlations among body composition variables were consistent across sex. We found a moderate inverse correlation between visceral adipose tissue index and density, suggesting that patients with more visceral adipose tissue have lower radiodensity quantities (i.e., indices). We also found a moderate positive correlation between the radiodensities of visceral and subcutaneous adipose tissues, suggesting that the densities of adipose tissue depots are similar.

To our knowledge, our study is the first to consider all six body composition variables together in a multivariable model to estimate associations between pre‐surgical body composition and pathological tumour features. Prior studies that investigated body composition in relation to stage and grade among patients with localized disease focused predominantly on quantity as opposed to the radiodensity of skeletal muscle, visceral adipose and subcutaneous adipose tissues, and included different RCC histologies. Consistent with some reports,^9^, ^10^, ^11^, ^12^, ^13^, ^16^ we initially detected positive associations for skeletal muscle index and visceral adipose tissue index in relation to stage and grade. However, after accounting for the radiodensities of these tissues in multivariable models, effect sizes were attenuated and became non‐significant. It is possible that prior studies may also have identified the radiodensities of skeletal muscle and visceral adipose tissue as more strongly associated with stage and grade or survival had they included all body composition variables in the multivariable models. Our findings highlight that both the index and radiodensity of all three tissue types are needed to gain a more complete understanding of how they are related to tumour aggressiveness at the time of surgery.

Strengths of our study include the large sample size, strict eligibility criteria, single histology, continuous variable assessment, simultaneous consideration of multiple body composition variables, and analyses that considered the impact of early age at onset, contrast enhanced CTs, and grade reclassification. We acknowledge the limitations that include the lack of racial and ethnic diversity of our study population, and smaller sample size of female participants. In addition, given the timespan of the Resolve Cohort (2000–2020), Fuhrman grade was used as a primary outcome because the ISUP grade was only available for the 114 patients who underwent nephrectomy after 2018. However, results from our exploratory probabilistic sensitivity analysis where we estimated ISUP for patients lacking this information produced similar results. Detailed information on other factors that influence body composition such as physical activity and diet were not available. However, the goal of this analysis was to identify a non‐invasive proxy for tumour aggressiveness that could be objectively and non‐invasively assessed prior to treatment selection, it was not an etiologic investigation of body composition. Because physical activity and diet influence body composition are upstream contributors to body composition but are not established risk factors for kidney cancer or stage and grade, they do not satisfy the criteria for confounding and do not need to be included in multivariable models. Finally, intramuscular adiposity was excluded from this report; it was not included independently nor as part of the other adiposity compartments. We chose not to include it given that it is the smallest of the body composition compartments and inter‐rater reliability is poor. Thus, we focused on the more robust measurements of body composition.

Our study suggests that radiodensities of skeletal muscle and visceral adipose tissues are novel, non‐invasive proxies for advanced tumour features and highlights the importance of evaluating the full range of body composition features simultaneously in multivariable models. Interpreting pre‐surgical CTs for body composition features may be a non‐invasive way to characterize patients prior to treatment selection, which is particularly important as renal biopsies are not routinely performed and active surveillance may be a safe strategy for patients with indolent renal masses.^41^ Moreover, because body composition variables are potentially modifiable,^42^, ^43^, ^44^ they may represent novel therapeutic targets. Future studies that investigate if pre‐surgical CT‐derived body composition features improve risk stratification for patients with renal masses and evaluate how baseline and changes in body composition after nephrectomy are associated with clinical outcomes are warranted.

## Conflict of interest statement

Helena Furberg, Patrick T. Bradshaw, Andrea Knezevic, Linnea Olsson, Stacey Petruzella, Emily Stein, Mike Paris, Jessica Scott, Oguz Akin, A. Ari Hakimi, Paul Russo, Alejandro Sanchez, Bette Caan, and Marina Mourtzakis declare they have no conflict of interest.

## Supporting information


**Figure S1.** Correlation matrix of body composition variables for males.
**Figure S2.** Correlation matrix of body composition variables for females.
**Table S1.** Associations between each body composition variable and advanced pathological characteristics in the Resolve Study showing the effect of contrast enhanced CTs.
**Table S2.** Age‐stratified* adjusted odds ratios (ORs) and 95% confidence intervals (CI) for associations between body composition variables and advanced stage and high grade in the Resolve Cohort.
**Table S3.** Associations between each body composition variable and high grade when patients are classified as Fuhrman vs. *estimated* International Society of Urologic Pathology (ISUP) grade.
