# Supplementary material for: Skeletal muscle and visceral adipose radiodensities are pre‐surgical, non‐invasive markers of aggressive kidney cancer
Source: J Cachexia Sarcopenia Muscle. 2024 Jan 24;15(2):726–34. doi: 10.1002/jcsm.13429 (PMC10995262; doi:10.1002/jcsm.13429)
Supplement: Supplementary file 1 — Figure S1. Correlation matrix of body composition variables for males. Figure S2. Correlation matrix of body composition variables for females. Table S1. Associations between each body composition variable and advanced pathological characteristics in the Resolve Study showing the effect of contrast enhanced CTs. Table S2. Age‐stratified* adjusted odds ratios (ORs) and 95% confidence intervals (CI) for associations between body composition variables and advanced stage and high grade in the Resolve Cohort. Table S3. Associations between each body composition variable and high grade when patients are classified as Fuhrman vs. estimated International Society of Urologic Pathology (ISUP) grade. [file JCSM-15-726-s001.docx]

**SUPPLEMENTARY MATERIALS**

**Supplemental Figure 1. Correlation matrix of body composition variables for males.**

**Supplemental Figure 2. Correlation matrix of body composition variables for females.**

**.**

**Supplemental Table 1. Associations between each body composition variable and advanced pathological characteristics in the Resolve Study showing the effect of contrast enhanced CTs**

|  | **ORs (95% CIs)** | | |
| --- | --- | --- | --- |
|  | **Including contrast enhanced CTs*** | **Excluding contrast enhanced CTs** | **Adjusted for contrast enhanced CTs** |
| **STAGE (Stage 3 vs. Stages 1-2) n=1239** | | | |
| **SMI per -10.99 cm^2^/m^2^** | 1.09 (0.90-1.32) | 1.07 (0.86-1.33) | 1.09 (0.90-1.32) |
| **SMD per -8.40 HU** | 1.61 (1.34-1.93) | 1.68 (1.37-2.07) | 1.62 (1.35-1.95) |
| **SATI per 46.57 cm^2^/m^2^** | 0.92 (0.76-1.10) | 0.88 (0.72-1.07) | 0.91 (0.76-1.10) |
| **SATD per 7.22 HU** | 0.90 (0.74-1.10) | 0.82 (0.65-1.02) | 0.91 (0.74-1.11) |
| **VATI per 35.26 cm^2^/m^2^** | 1.19 (0.95-1.50) | 1.11 (0.86-1.42) | 1.18 (0.94-1.48) |
| **VATD per 7.61 HU** | 1.41 (1.10-1.81) | 1.40 (1.05-1.85) | 1.38 (1.07-1.79) |
|  |  |  |  |
| **FUHRMAN GRADE (Grades 3-4 vs. Grades 1-2) n=1125** | | | |
| **SMI per -10.99 cm^2^/m^2^** | 1.04 (0.86-1.26) | 0.99 (0.81-1.21) | 1.04 (0.86-1.26) |
| **SMD per -8.40 HU** | 1.54 (1.28, 1.86) | 1.43 (1.17-1.74) | 1.54 (1.28-1.86) |
| **SATI per 46.57 cm^2^/m^2^** | 0.93 (0.78-1.11) | 0.91 (0.76-1.10) | 0.93 (0.78-1.11) |
| **SATD per 7.22 HU** | 0.91 (0.75-1.11) | 0.89 (0.73-1.10) | 0.91 (0.75-1.12) |
| **VATI per 35.26 cm^2^/m^2^** | 1.10 (0.88-1.38) | 1.22 (0.96-1.54) | 1.10 (0.87-1.38) |
| **VATD per 7.61 HU** | 1.36 (1.07-1.74) | 1.45 (1.12-1.88) | 1.36 (1.06-1.75) |
|  |  |  |  |

* OR (95% CI): Odds ratio and 95% confidence interval compares each body composition variable in relation to advanced stage (Stage II vs. Stages I-II) and high grade (grade 3-4 vs. grades 1-2). Adjusted for age, sex, and all body composition variables. SMI: Skeletal Muscle Index; SMD: Skeletal Muscle Density; VATI: Visceral Adipose Tissue Index; VATD: Visceral Adipose Tissue Density; SATI: Subcutaneous Adipose Tissue Index; SATD: Subcutaneous Adipose Tissue Density; HU: Hounsfield Units. Global test of all body composition-contrast interaction: Likelihood ratio test p-value 0.12 for Stage; 0.70 for Grade.

**Supplementary Table 2** Age-stratified* adjusted odds ratios (ORs) and 95% confidence intervals (CI) for associations between body composition variables and advanced stage and high grade in the Resolve Cohort

|  | **Fully Adjusted**  **OR (95% CI)**  **Early onset ccRCC*** | **Fully Adjusted**  **OR (95% CI)**  **Later onset ccRCC*** | **Interaction test p-value** |
| --- | --- | --- | --- |
| **STAGE (Stage 3 vs. Stages 1-2) n=1239** | | | |
| **SMI per -10.99 cm^2^/m^2^** | 0.93 (0.59-1.47) | 1.13 (0.91-1.39) | 0.449 |
| **SMD per -8.40 HU** | 1.76 (0.98-3.19) | 1.58 (1.30-1.92) | 0.725 |
| **SATI per 46.57 cm^2^/m^2^** | 0.96 (0.61-1.43) | 0.90 (0.74-1.10) | 0.806 |
| **SATD per 7.22 HU** | 0.93 (0.53-1.59) | 0.88 (0.71-1.09) | 0.856 |
| **VATI per 35.26 cm^2^/m^2^** | 1.04 (0.50-2.11) | 1.23 (0.97-1.57) | 0.653 |
| **VATD per 7.61 HU** | 1.30 (0.68-2.47) | 1.46 (1.11-1.92) | 0.745 |
|  | | | |
| **FUHRMAN GRADE (Grades 3-4 vs. Grades 1-2) n=1125** | | | |
| **SMI per -10.99 cm^2^/m^2^** | 0.97 (0.64-1.46) | 1.09 (0.88-1.34) | 0.629 |
| **SMD per -8.40 HU** | 1.31 (0.75-2.30) | 1.57 (1.29-1.92) | 0.541 |
| **SATI per 46.57 cm^2^/m^2^** | 0.90 (0.59-1.33) | 0.93 (0.77-1.13) | 0.894 |
| **SATD per 7.22 HU** | 1.05 (0.64-1.72) | 0.89 (0.72-1.11) | 0.565 |
| **VATI per 35.26 cm^2^/m^2^** | 1.27 (0.67-2.46) | 1.09 (0.86-1.40) | 0.669 |
| **VATD per 7.61 HU** | 1.45 (0.82-2.59) | 1.36 (1.04-1.80) | 0.848 |

*Early onset defined as <46 years of age at the time of diagnosis based on Shuch et al.

* OR (95% CI): Odds ratio and 95% confidence interval compares each body composition variable in relation to advanced stage (Stage II vs. Stages I-II) and high grade (grade 3-4 vs. grades 1-2). Adjusted for age, sex, and all body composition variables. SMI: Skeletal Muscle Index; SMD: Skeletal Muscle Density; VATI: Visceral Adipose Tissue Index; VATD: Visceral Adipose Tissue Density; SATI: Subcutaneous Adipose Tissue Index; SATD: Subcutaneous Adipose Tissue Density; HU: Hounsfield Units. Global test of all body composition-age interaction: Likelihood ratio test p-value 0.99 for Stage; 0.80 for Grade.

**Probabilistic Sensitivity Analysis of Grade Reclassification Analysis**

**Supplementary Table 3. Associations between each body composition variable and high grade when patients are classified as Fuhrman vs. *estimated* International Society of Urologic Pathology (ISUP) grade.**

|  | **Adjusted OR (95% CI)** | |
| --- | --- | --- |
|  | **Fuhrman Grade** | **Estimated ISUP Grade** |
| **SMI per -10.99 cm^2^/m^2^** | 1.04 (0.86-1.26) | 1.01 (0.81-1.27) |
| **SMD per -8.40 HU** | **1.54 (1.28-1.86)** | **1.25 (1.01-1.55)** |
| **SATI per 46.57 cm^2^/m^2^** | 0.93 (0.78-1.11) | 0.94 (0.75-1.17) |
| **SATD per 7.22 HU** | 0.91 (0.75-1.11) | 0.96 (0.75-1.21) |
| **VATI per 35.26 cm^2^/m^2^** | 1.10 (0.88-1.38) | 1.15 (0.86-1.51) |
| **VATD per 7.61 HU** | **1.36 (1.07-1.74)** | **1.29 (0.97-1.75)** |

* Odds ratios (ORs) and 95% confidence intervals Adjusted for age, sex, and all body composition variables. SMI: Skeletal Muscle Index; SMD: Skeletal Muscle Density; VATI: Visceral Adipose Tissue Index; VATD: Visceral Adipose Tissue Density; SATI: Subcutaneous Adipose Tissue Index; SATD: Subcutaneous Adipose Tissue Density; HU: Hounsfield Units

**Methods**

At our institution, the grading system changed from Fuhrman to ISUP for surgeries occurring on/after January 1, 2018. Our cohort is comprised of patients who had nephrectomies between 2000 and 2020. Our primary analysis presents results limited to the 1,125 patients with Furhman grade, which is the majority of our cohort. However, since ISUP is the current standard, we performed a sensitivity analysis of outcome misclassification to characterize the robustness of our findings to the new grading system. For the 1125 patients who were diagnosed before January 1, 2018, we randomly generated an ISUP grade given their observed Fuhrman grade.^^[[1]](#footnote-1)^^ Probabilities for ISUP classification were derived from the cell counts cross-classifying ISUP and Fuhrman grade reported in Table III of Odeh et al^2^. Within each level of Fuhrman grade, the counts for each ISUP category were divided by the Fuhrman-specific total. For example, a patient with Fuhrman grade 1, had a 63% probability (=34/54) of being assigned ISUP grade 1, a 31% probability (=17/54) of ISUP grade 2, and 6% probability (=3/54) of ISUP grade 3 (no probability of ISUP grade 4). For the 114 patients with date of nephrectomy on/after January 1, 2018 their observed ISUP grade was used.

|  | ISUP=1 | ISUP=2 | ISUP=3 | ISUP=4 |
| --- | --- | --- | --- | --- |
| Fuhrman=1 | 0.63 | 0.31 | 0.06 | 0 |
| Fuhrman=2 | 0.3 | 0.57 | 0.13 | 0.01 |
| Fuhrman=3 | 0.01 | 0.43 | 0.49 | 0.08 |
| Fuhrman=4 | 0 | 0.05 | 0.22 | 0.73 |

After the imputation step, the 4-level ISUP grades were dichotomized into high (3 and 4) vs. low (1 and 2) grade. This dichotomous variable was used as the outcome in a logistic regression model as described in the main text, including all body composition measures, adjusted for age (continuous) and biological sex. As a final step, to account for sampling variability the logistic regression model parameters were re-sampled from a multivariate normal distribution with mean and covariance matrix parameters given by the vector of point estimates and estimated covariance matrix, respectively.^3^ The sampled parameters for the body composition characteristics were transformed to ORs. This process (starting at the imputation of ISUP grade) was repeated 2000 times, accumulating the ORs from each iteration. The 2000 ORs for each body composition variable were summarized with the median and 2.5% and 97.5% quantiles (corresponding to a 95% simulation-based confidence interval^3^).

The table below shows the **Sensitivity Analysis Results** which includes all 1,239 patients, but with 1,125 with Fuhrman grade (diagnosed before 2018) randomly re-assigned to ISUP grade based on their observed Fuhrman status.

Across 2000 simulations, the median proportion of the 1125 patients with Fuhrman grade who were initially classified as low Fuhrman grade (1 or 2) and reclassified as high ISUP grade (3 or 4) was 0.07, those with high Fuhrman grade reclassified as low ISUP grade was 0.19, and those with high or low Fuhrman who remained the same was 0.74.Generally, the probabilistic re-classification of Fuhrman to ISUP grade resulted in qualitatively similar, but slightly attenuated associations. After re-classification, the SMD association remained notable (corrected OR (95% CI): 1.25 (1.01, 1.55)), as did VATD (corrected OR (95% CI): 1.29 (0.97, 1.75)). Confidence intervals are slightly wider for the sensitivity analysis as the uncertainty in the re-classification step is propagated through to these estimates.

**References**

1. Fox, M. P., MacLehose, R. F. & Lash, T. L. *Applying quantitative bias analysis to epidemiologic data*. (Springer, 2022).

2. Odeh, S. *et al.* Histologic re-evaluation of a population-based series of renal cell carcinomas from The Netherlands Cohort Study according to the 2022 ISUP/WHO classification. *Oncology Letters* **25**, 174 (2023).

3. Greenland, S. Interval estimation by simulation as an alternative to and extension of confidence intervals. *Int J Epidemiol* **33**, (2004).

1. [↑](#footnote-ref-1)
